# To Test or to Treat? An Analysis of Influenza Testing and Antiviral Treatment Strategies Using Economic Computer Modeling

**DOI:** 10.1371/journal.pone.0011284

**Published:** 2010-06-23

**Authors:** Bruce Y. Lee, Sarah M. McGlone, Rachel R. Bailey, Ann E. Wiringa, Shanta M. Zimmer, Kenneth J. Smith, Richard K. Zimmerman

**Affiliations:** 1 Department of Medicine, School of Medicine, University of Pittsburgh, Pittsburgh, Pennsylvania, United States of America; 2 Department of Biomedical Informatics, School of Medicine, University of Pittsburgh, Pittsburgh, Pennsylvania, United States of America; 3 Department of Epidemiology, Graduate School of Public Health, University of Pittsburgh, Pittsburgh, Pennsylvania, United States of America; 4 Department of Family Medicine, University of Pittsburgh, Pittsburgh, Pennsylvania, United States of America; Yale University, United States of America

## Abstract

**Background:**

Due to the unpredictable burden of pandemic influenza, the best strategy to manage testing, such as rapid or polymerase chain reaction (PCR), and antiviral medications for patients who present with influenza-like illness (ILI) is unknown.

**Methodology/Principal Findings:**

We developed a set of computer simulation models to evaluate the potential economic value of seven strategies under seasonal and pandemic influenza conditions: (1) using clinical judgment alone to guide antiviral use, (2) using PCR to determine whether to initiate antivirals, (3) using a rapid (point-of-care) test to determine antiviral use, (4) using a combination of a point-of-care test and clinical judgment, (5) using clinical judgment and confirming the diagnosis with PCR testing, (6) treating all with antivirals, and (7) not treating anyone with antivirals. For healthy younger adults (<65 years old) presenting with ILI in a seasonal influenza scenario, strategies were only cost-effective from the societal perspective. Clinical judgment, followed by PCR and point-of-care testing, was found to be cost-effective given a high influenza probability. Doubling hospitalization risk and mortality (representing either higher risk individuals or more virulent strains) made using clinical judgment to guide antiviral decision-making cost-effective, as well as PCR testing, point-of-care testing, and point-of-care testing used in conjunction with clinical judgment. For older adults (≥65 years old), in both seasonal and pandemic influenza scenarios, employing PCR was the most cost-effective option, with the closest competitor being clinical judgment (when judgment accuracy ≥50%). Point-of-care testing plus clinical judgment was cost-effective with higher probabilities of influenza. Treating all symptomatic ILI patients with antivirals was cost-effective only in older adults.

**Conclusions/Significance:**

Our study delineated the conditions under which different testing and antiviral strategies may be cost-effective, showing the importance of accuracy, as seen with PCR or highly sensitive clinical judgment.

## Introduction

Although prompt antiviral treatment may be able to improve outcomes for adults infected by either seasonal or pandemic (such as novel H1N1) influenza viruses, antiviral treatment is costly, $77 to $121 per patient (due to repackaging differences). Antivirals may be particularly useful for older adults (≥65 years old), who are at greater risk for influenza complications [1]. Testing may help distinguish influenza from other types of influenza-like illness (ILI) [2]. Many clinicians use patient symptoms to identify those who may have influenza and benefit from a course of antiviral therapy. As with any test, clinical judgment is less than perfect and has varying degrees of accuracy [3], [4]. Testing for influenza, with either rapid influenza tests or polymerase chain reaction (PCR), may help better diagnose influenza and guide antiviral treatment [5]. However, these tests also have associated costs and less than perfect sensitivity and specificity. In fact, recent reports suggest that currently available rapid tests have relatively low sensitivity in detecting the novel influenza A (H1N1) strain [6], [7], [8], [9]. Finally, in pandemic scenarios, some clinicians may be inclined to administer antivirals to everyone presenting with ILI if they believe that morbidity and mortality risk are elevated.

Currently, no consensus exists over influenza testing of patients presenting with ILI in seasonal or pandemic influenza scenarios [10], [11]. The optimal approach will minimize expected associated costs while maximizing expected clinical effects, i.e., provide antiviral treatment to those who truly have influenza. Economic modeling can help address this ongoing question and assist clinicians in their decision making, third-party payors in their insurance coverage policies, test manufacturers in their pricing strategies, scientists in their test development, and public health officials in their policy making. Economic value can be particularly informative during an influenza pandemic when time is short, available resources may be limited, and outcomes may be worse.

We developed a computer simulation model to compare the potential economic impact of different testing and antiviral use strategies for patients presenting to the clinic or emergency room with ILI symptoms. Simulation runs examined both seasonal and pandemic influenza scenarios and explored the effects of varying the probability of a patient with ILI having influenza, test sensitivity and specificity, clinical judgment sensitivity, patient age, and the probability of influenza outcomes such as hospitalization and mortality. Additional scenarios explored the decision for higher-risk adults (i.e., double the risk of hospitalization and mortality), older adults, and higher-risk older adults.

## Methods

### Model Structures

**Figure 1:**
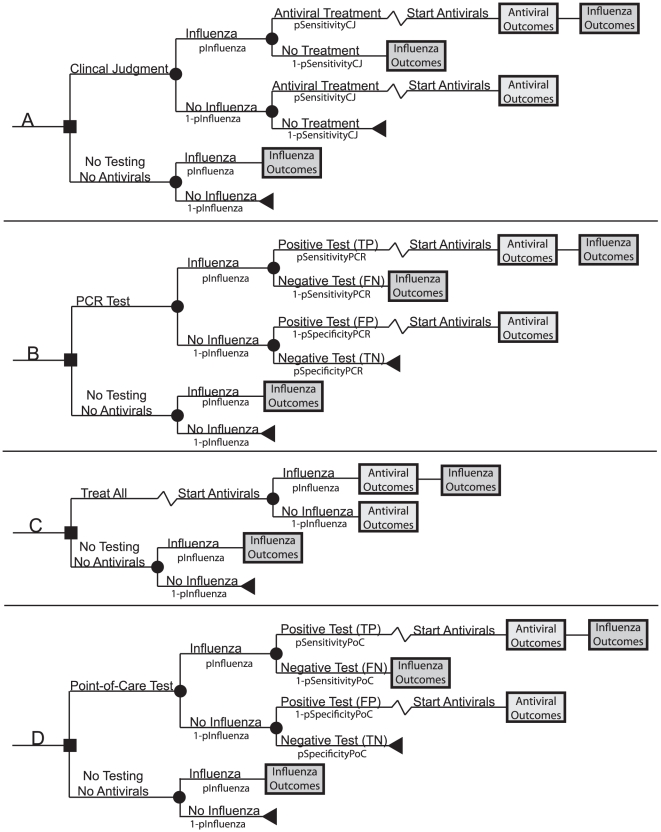
Influenza testing base structure. a) clinical judgment b) PCR testing c) antivirals to all d) point-of-care testing.

**Figure 2:**
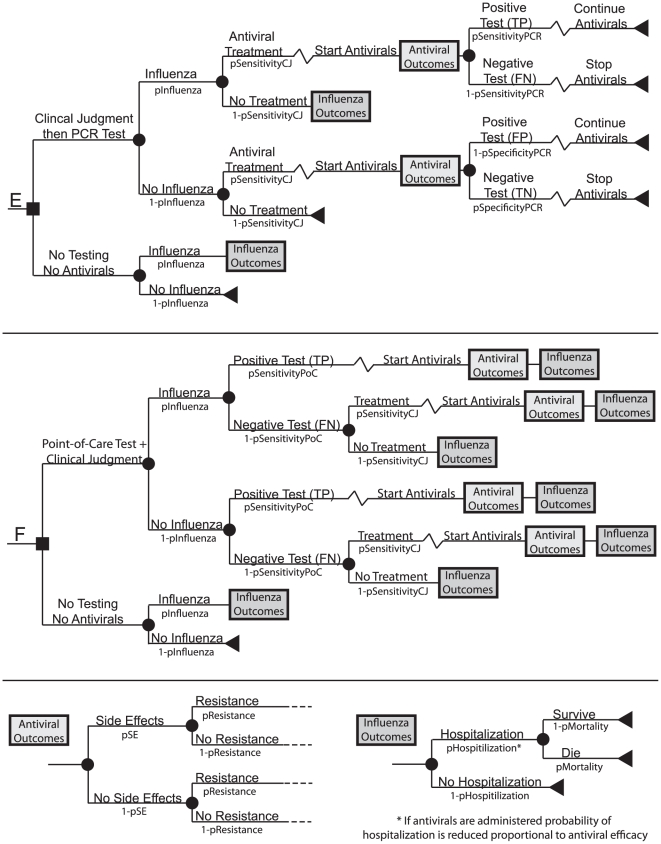
Influenza testing base structure. e) clinical judgment then PCR testing f) point-of-care testing with clinical judgment. Antiviral and influenza outcomes tree structures.

Figures 1 and 2 depict the general structure of our Monte Carlo decision analytic computer simulation models, constructed using TreeAge Pro 2009 (TreeAge Software, Williamstown, Massachusetts). Each simulation run for both the younger adults (ages 20 to 64) and older adults (ages 65 to 85) sent 5,000 simulated adults 5,000 times (i.e., 25,000,000 trials) through the model. These models represented an outpatient presenting to the clinic or emergency room with ILI and a clinician's choice among the following options:

- Clinical judgment alone to distinguish influenza from ILI to guide antiviral use.
- Clinical judgment to decide and then confirming with PCR testing.
- PCR test and treat if positive (for outpatient settings with PCR readily available).
- Rapid (point-of-care) test and treat if positive.
- Point-of-care test and treat if positive and if negative use clinical judgment to decide.
- Treat all patients with antivirals without testing (i.e., clinicians give antivirals to everyone presenting with ILI).
- No antiviral treatment.

Separate scenarios explored the decision from the third-party payor perspective (considering only direct costs of illness) and the societal prospective (considering direct and indirect costs).

ILI had a probability of being influenza. Test results were available in 24 hours (if test was available at time of visit) and incorporated their corresponding sensitivities and specificities. The effects of varying clinical judgment sensitivity (i.e., the ability of clinicians to immediately detect a case of influenza without utilizing tests) were explored. Antiviral treatment consisted of 75 mg of oseltamivir twice a day for five days and reduced the length of influenza illness, hospitalization risk, and mortality. Patients who received antivirals had a probability of side effects [mainly gastrointestinal with attendant quality-adjusted life year (QALY) decrements]. Additionally, there was a probability of antiviral resistance. All patients who did not receive antivirals or require hospitalization, self-treated with over-the-counter medications.

The following equation calculated the incremental cost-effectiveness ratio (ICER) of each strategy versus the comparator (i.e., not giving anyone antiviral medications):
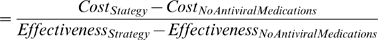
Our model measured effectiveness in QALYs. A strategy was considered cost-effective if the ICER was less than $50,000 per quality-adjusted life-year (QALY).

### Data Inputs

Table 1 lists the various data inputs for our model and the corresponding distributions and data sources used. We used triangular distributions for all of our utility variables and gamma, beta, or triangular distributions for all other variables. For variables which may have skewed distributions, such as costs, gamma distributions were used [12]. For probabilities that approximated normal distributions, we employed beta distributions which are bounded by 0 and 1, unlike normal distributions which can generate values outside this interval [13]. When limited data existed providing only the lower limit, and upper limit of a variable's value, we utilized triangular distributions. Where possible, data inputs came from published meta-analyses. All costs were in 2009 U.S. dollars, a 3% discount rate converted all costs into 2009 values. Our model measured effectiveness in QALYs. A healthy person accrued the total complement of their age-adjusted QALYs. Influenza and hospitalization each caused different decrements in QALYs accrued for their durations. Patients who did not survive lost QALYs based on their quality-adjusted life expectancies derived from the Human Mortality Database [14]. These future life-years were discounted by 3% per year.

**Table 1:** Data inputs for model variables.

Description (units)	Variable Name in Figures	Dis*	Mean	Standard Deviation	Range	Source
**COSTS ($US)**						
Neuraminidase Inhibitor		γ	99.32	21.99		[26]
Clinic Visit		Δ	104.77		69.14–140.70	[27]
Median Hourly Wage			16,52			[28]
Over the Counter Medications		Δ	15.61		11.70–19.51	[26]
Hospitalization, 18–44 yrs		γ	3,643.13	785.07		[29]
Hospitalization, 45–64 yrs		γ	4,396.37	1,354.77		[29]
Hospitalization, 65–84 yrs		γ	5,332.08	528.32		[29]
Death in Hospital			5,000		-	[30]
PCR Test			29		-	Expert Opinion
Rapid Test			22		-	Expert Opinion
**DURATIONS (days)**						
Influenza			7		-	
Time Missed from Work		Δ	3.2		1.5–4.9	[31]
Time Antivirals Reduce Symptoms		Δ	1.4		1.0–2.0	[32]
**UTILITIES (QALYs)**						
One Year of Life for Adults, 18–64 yrs			0.92		-	[33]
One Year of Life for Adults, 65–85 yrs			0.84		-	[33]
***Utility/Day***						
Influenza-Like Illness (ILI)		Δ	0.725		0.61–0 .84	[34], [35]
Influenza no Hospitalization		Δ	0.5956		0.5579–0.65	[30], [36], [37], [38], [39], [40], [41]
Influenza with Hospitalization		Δ	0.40		0.38–0.50	[37], [40], [41]
Antiviral Side Effects		Δ	0.835		0.77–0.90	[30], [42]
**PROBABILITIES**						
Antiviral Side Effects	pSE	β	0.126	0.0440		[43], [44], [45]
Antiviral Resistance	pResistance	Δ	0.02		0.004–0.05	[32], [46], [47], [48], [49]
Hospitalization Given Influenza, 65–84 yrs	pHospitalization	Δ	0.04		0.01–0.07	[1]
Hospitalization Given Influenza, 18–54 yrs	pHospitalization	Δ	0.004		0.001–0.007	[50]
Antiviral Efficacy in Reducing Hospitalization		Δ	0.78		0.00–0.98	[18], [19]
Influenza Mortality, 18–44 yrs	pMortality		0.0105		-	[29]
Influenza Mortality, 45–64 yrs	pMortality		0.0235		-	[29]
Influenza Mortality, 65–85 yrs	pMortality		0.0441		-	[29]
**SENSITIVITY ANALYSIS**			**Sensitivity Analysis Values**			
ILI being Influenza	pInfluenza		0.10, 0.20, 0.20			[51]
Clinical Judgment Sensitivity	pSensitivityCJ		0.25, 0.50, 0.75			[52]
PCR Sensitivity	pSensitivityPCR		0.90, 0.95			[9], [52], [53]
PCR Specificity	pSpecificityPCR		0.95, 1.00			[9], [52], [53]
Point of Care Sensitivity	pSensitivityPoC		0.25, 0.50, 075			[6], [7], [8], [9], [52], [54]
Point of Care Specificity	pSpecificityPoC		0.90, 0.95			[6], [7], [8], [9], [52], [54]

*Distribution Type: γ = gamma, β = beta, Δ = triangular.

### Sensitivity Analyses

Sensitivity analyses determined the effects of varying different parameter values individually throughout the ranges listed in Table 1. Multi-dimensional sensitivity analyses were performed on selected parameters. In particular, we examined the effects of varying PCR test sensitivity (90%, 95%) and specificity (95%, 100%), the sensitivity (25%, 50%, 75%) and specificity (90%, 95%) of the point-of-care test, and the sensitivity of clinical judgment (25%, 50%, 75%) to represent differences in test performance in both seasonal and pandemic influenza conditions. To understand how results may change with more virulent circulating influenza virus strain (twice as virulent) or higher risk patients (twice as prone to hospitalization or death), sensitivity analyses varied the probability of hospitalization and mortality from influenza (respectively, up to two times that of seasonal influenza). Since the true increased risk of hospitalization and death may be highly variable under these circumstances, this sensitivity analysis was done due to the actual probabilities of hospitalization and mortality of pandemic influenza being unknown. The probability of ILI being influenza was varied from 10% to 20% to 30%. In addition, probabilistic (Monte Carlo) sensitivity analyses examined the effects of varying all parameters along their possible ranges.

## Results

### Seasonal Influenza Scenarios with Baseline Morbidity and Mortality

Table 2 (societal perspective) and Table S1 (third-party payor perspective) show the ICER of each strategy versus the control (no antiviral medications for any patients) among younger adults (ages 20 to 64) for seasonal influenza scenarios. These results include sensitivity analyses varying the sensitivity and specificity of different testing strategies. In general, simulation runs suggested that routinely using antivirals was not cost-effective (i.e., ICER was greater than $50,000/QALY) in younger adults, even when guided by testing or clinical judgment from the third party payor perspective. In Table 2, the situations where the ICER was less than $50,000/QALY from the societal perspective are designated in bold.

**Table 2:** Incremental cost-effectiveness ratios (in $US per quality-adjusted life-years) of different approaches to patients aged 20 to 64 years with influenza-like illness (ILI) from the societal perspective for seasonal influenza.

	Probability of ILI being Influenza		
Strategy	10%	20%	30%
*Baseline Seasonal Influenza Hospitalization Risk and Mortality*			
Treat all with Antivirals	Do Nothing	255,981–271,024	61,287–65,255
Clinical Judgment (25)†	Do Nothing	Do Nothing	1,350,402–1,792,375
Clinical Judgment (50)	Do Nothing	286,577–290,692	53,840–59,494
Clinical Judgment (75)	131,522–201,789	***Dominant***	***Dominant***
PCR Test (90/95)*	134,800–146,777	**32,320–42,414**	**1,555–2,157**
PCR Test (90/100)	115,838–123,300	**22,079–25,240**	***Dominant***
PCR Test (95/100)	103,145–104,566	**18,363–21,762**	***Dominant***
PCR Test (90/95)+CJ (25)	Do Nothing	541,092–634,618	149,340–239,616
PCR Test (90/95)+CJ (50)	Do Nothing	612,506–841,518	182,798–263,160
PCR Test (90/95)+CJ (75)	Do Nothing	549,754–1,356,977	162,449–206,521
PCR Test (90/100)+CJ (25)	Do Nothing	512,980–711,987	131,079–163,658
PCR Test (90/100)+CJ (50)	Do Nothing	434,991–771,128	182,643–198,933
PCR Test (90/100)+CJ (75)	Do Nothing	543,776–591,240	169,910–190,378
PCR Test (95/100)+CJ (25)	Do Nothing	667,556–704,636	103,596–142,230
PCR Test (95/100)+CJ (50)	Do Nothing	430,605–515,751	143,424–157,084
PCR Test (95/100)+CJ (75)	Do Nothing	449,201–681,528	143,583–156,729
Point-of-Care Test (25/95)	625,601–1,039,207	193,685–234,868	120,186–124,282
Point-of-Care Test (50/95)	178,094–215,502	72,209–76,111	**26,303–28,149**
Point-of-Care Test (75/95)	108,820–126,429	**27,469–33,194**	***Dominant***
Point-of-Care Test (25/95)+CJ (25)	Do Nothing	3,392,605–3,474,515	333,795–534,802
Point-of-Care Test (25/95)+CJ (50)	Do Nothing	330,944–334,942	103,681–127,920
Point-of-Care Test (25/95)+CJ (75)	314,229–453,120	79,798–108,930	**25,276–29,208**
Point-of-Care Test (50/95)+CJ (25)	Do Nothing	717,676–1,026,360	201,643–256,826
Point-of-Care Test (50/95)+CJ (50)	Do Nothing	207,952–213,952	75,563–77,585
Point-of-Care Test (50/95)+CJ (75)	253,632–382,295	70,983–79,551	**18,662–21,233**
Point-of-Care Test (75/95)+CJ (25)	Do Nothing	320,955–408,254	111,650–114,703
Point-of-Care Test (75/95)+CJ (50)	1,463,398–3,226,593	157,730–166,551	54,372–56,919
Point-of-Care Test (75/95)+CJ (75)	306,082–355,297	62,538–73,435	**11,731–14,727**

Comparator: Do nothing.

† (Sensitivity).

*(Sensitivity/Specificity).

Bold Text: Strategy is cost effective (ICER versus Do Nothing is <$50,000 per QALY).

Bold and Italic Text: Strategy is economically dominant (costs less and is more effective than Do Nothing).

The Table 3 (societal perspective) and Table S2 (third-party payor perspective) show the ICER of each strategy versus the control (no antiviral medications for any patients) for older adults (65+ years) in baseline seasonal influenza scenarios. As can be seen, many of the testing strategies become cost-effective especially when the probability of ILI being influenza increases to 20% and 30%.

**Table 3:** Incremental cost-effectiveness ratios (in $US per quality-adjusted life-years) of different approaches to patients aged 65 to 85 years with influenza-like illness (ILI) from the societal perspective for seasonal influenza.

	Probability of ILI being Influenza		
Strategy	10%	20%	30%
*Baseline Seasonal Influenza Hospitalization Risk and Mortality*			
Treat all with Antivirals	60,028–84,119	**22,841–33,040**	**11,783–16,158**
Clinical Judgment (25)†	285,620–421,268	92,675–151,473	51,643–62,050
Clinical Judgment (50)	64,445–96,812	**22,952–29,547**	**11,589–16,857**
Clinical Judgment (75)	**15,611–21,345**	**5,135–6,963**	**1,400–2,396**
PCR Test (90/95)*	**22,282–30,188**	**10,377–13,514**	**6,112–6,899**
PCR Test (90/100)	**19,872–28,254**	**9,315–11,795**	**4,823–6,406**
PCR Test (95/100)	**18,892–25,540**	**8,283–10,859**	**4,526–5,519**
PCR Test (90/95)+CJ (25)	97,191–122,508	41,190–52,291	**21,682–34,376**
PCR Test (90/95)+CJ (50)	112,567–151,452	37,727–53,910	**23,402–29,228**
PCR Test (90/95)+CJ (75)	103,131–146,857	40,487–58,018	**22,282–29,423**
PCR Test (90/100)+CJ (25)	83,722–130,766	**41,423–44,423**	**23,790–29,753**
PCR Test (90/100)+CJ (50)	92,260–121,667	38,334–54,178	**23,272–30,098**
PCR Test (90/100)+CJ (75)	102,150–139,094	38,725–53,723	**21,938–31,056**
PCR Test (95/100)+CJ (25)	71,334–114,795	**32,281–51,579**	**21,760–28,657**
PCR Test (95/100)+CJ (50)	87,555–130,347	**35,535–46,654**	**21,642–28,646**
PCR Test (95/100)+CJ (75)	87,265–126,752	**37,490–50,604**	**21,723–27,774**
Point-of-Care Test (25/95)	86,911–88,159	**30,347–37,452**	**24,732–26,032**
Point-of-Care Test (50/95)	**38,060–48,071**	**16,708–21,038**	**8,795–12,718**
Point-of-Care Test (75/95)	**22,367–30,731**	**9,280–13,839**	**4,733–6,090**
Point-of-Care Test (25/95)+CJ (25)	188,184–299,894	68,453–89,056	**36,948–49,960**
Point-of-Care Test (25/95)+CJ (50)	87,471–110,599	**32,429–44,525**	**17,936–23,989**
Point-of-Care Test (25/95)+CJ (75)	**43,839–50,862**	**16,911–21,976**	**8,768–11,795**
Point-of-Care Test (50/95)+CJ (25)	124,841–148,754	42,529–57,340	**23,804–31,963**
Point-of-Care Test (50/95)+CJ (50)	61,417–92,954	**25,539–33,208**	**14,557–19,005**
Point-of-Care Test (50/95)+CJ (75)	**34,915–49,613**	**14,748–19,180**	**7,940–10,148**
Point-of-Care Test (75/95)+CJ (25)	85,786–118,320	**33,122–42,735**	**18,404–23,233**
Point-of-Care Test (75/95)+CJ (50)	58,798–73,172	**21,361–30,208**	**11,958–16,030**
Point-of-Care Test (75/95)+CJ (75)	**32,920–42,902**	**13,268–17,098**	**7,130–9,215**

Comparator: Do nothing.

† (Sensitivity).

*(Sensitivity/Specificity).

Bold Text: Strategy is cost effective (ICER versus Do Nothing is <$50,000 per QALY).

Bold and Italic Text: Strategy is economically dominant (costs less and is more effective than Do Nothing).

### Pandemic Influenza (More Severe Influenza Virus Strain) or High Risk Patients (Higher Morbidity and Mortality)

Additional scenarios explored the effects of doubling influenza-attributable hospitalization and death risks, which would correspond to either a more severe influenza strain or a higher-risk patient. Table 4 and Table S1 (lower half) show the ICERs of each strategy versus the control (no antiviral medications for any patients) for younger adults (ages 20 to 64). Using clinical judgment (sensitivity ≥75%) to guide antiviral treatment emerged as the most cost-effective option when the probability of influenza was ≥10%. The closest competitor to clinical judgment was PCR testing, followed by point-of-care testing.

**Table 4:** Incremental cost-effectiveness ratios (in $US per quality-adjusted life-years) of different approaches to patients aged 20 to 64 years with influenza-like illness (ILI) from the societal perspective for pandemic influenza or high risk patients.

	Probability of ILI being Influenza		
Strategy	10%	20%	30%
*Pandemic or High Risk Patients (2x Seasonal Influenza Hospitalization Risk and Mortality)*			
Treat all with Antivirals	344,799–592,966	60,250–84,750	**17,901–23,898**
Clinical Judgment (25)	Do Nothing	390,342–789,151	160,149–373,427
Clinical Judgment (50)	269,233–411,339	75,155–81,362	**18,668–25,379**
Clinical Judgment (75)	**37,503–45,934**	***Dominant***	***Dominant***
PCR Test (90/95)	62,190–63,018	**12,819–16,495**	***Dominant***
PCR Test (90/100)	50,400–51,477	**7,847–10,767**	***Dominant***
PCR Test (95/100)	**43,512–43,801**	**6,072–8,740**	***Dominant***
PCR Test (90/95)+CJ (25)	422,205–688,019	139,829–172,092	62,417–65,315
PCR Test (90/95)+CJ (50)	676,451–2,876,402	138,226–140,685	51,234–86,507
PCR Test (90/95)+CJ (75)	986,507–1,234,361	126,403–184,271	57,336–74,509
PCR Test (90/100)+CJ (25)	547,979–774,875	109,323–292,613	50,547–55,795
PCR Test (90/100)+CJ (50)	735,287–1,797,558	122,160–181,200	52,694–78,051
PCR Test (90/100)+CJ (75)	935,033–1,054,990	125,255–161,079	59,849–68,330
PCR Test (95/100)+CJ (25)	249,055–575,055	148,139–152,896	59,7356–74,683
PCR Test (95/100)+CJ (50)	581,611–1,407,688	129,831–140,404	43,852–56,130
PCR Test (95/100)+CJ (75)	429,612–519,042	123,864–142,654	50,019–70,622
Point-of-Care Test (25/95)	166,899–242,218	87,611–138,701	51,993–56,218
Point-of-Care Test (50/95)	90,359–130,079	**27,887–36,463**	**8,827–10,913**
Point-of-Care Test (75/95)	51,637–66,850	**10,189–12,618**	***Dominant***
Point-of-Care Test (25/95)+CJ (25)	Do Nothing	236,096–384,279	156,413–171,915
Point-of-Care Test (25/95)+CJ (50)	381,699–416,699	103,613–105,420	40,661–53,021
Point-of-Care Test (25/95)+CJ (75)	102,697–168,367	**33,071–40,082**	**7,576–9,994**
Point-of-Care Test (50/95)+CJ (25)	988,213–1,909,470	154,926–183,070	69,634–73,471
Point-of-Care Test (50/95)+CJ (50)	231,581–310,961	69,986–75,760	**26,809–37,315**
Point-of-Care Test (50/95)+CJ (75)	110,861–134,020	**26,833–33,309**	**4,996–7,811**
Point-of-Care Test (75/95)+CJ (25)	361,604–761,138	97,237–106,912	**39,757–47,102**
Point-of-Care Test (75/95)+CJ (50)	214,597–261,459	56,607–69,742	**18,622–23,655**
Point-of-Care Test (75/95)+CJ (75)	93,714–109,034	**22,917–29,311**	**3,028–4,733**

Comparator: Do nothing.

† (Sensitivity).

*(Sensitivity/Specificity).

Bold Text: Strategy is cost effective (ICER versus Do Nothing is <$50,000 per QALY).

Bold and Italic Text: Strategy is economically dominant (costs less and is more effective than Do Nothing).

Table 5 and Table S2 (lower half) show the ICERs of each strategy versus the control (no antiviral medications for any patients) for scenarios in which influenza hospitalization risk and mortality were double that of seasonal influenza for older adults (65+ years old). All strategies were found to be cost-effective, except clinical judgment (25% sensitive) when the probability of influenza was 20%. Employing PCR to guide antiviral initiation emerged as the most cost-effective option, becoming dominant for most conditions. The closest competitor to PCR was clinical judgment, followed by point-of-care testing, point-of-care testing in combination with clinical judgment, and clinical judgment confirmed by PCR testing.

**Table 5:** Incremental cost-effectiveness ratios (in $US per quality-adjusted life-years) of different approaches to patients aged 65 to 85 years with influenza-like illness (ILI) from the societal perspective for pandemic influenza or high risk patients.

	Probability of ILI being Influenza		
Strategy	10%	20%	30%
*Pandemic or High Risk Patients (2x Seasonal Influenza Hospitalization Risk and Mortality)*			
Treat all with Antivirals	**11,320–15,765**	**3,076–4,227**	**318–467**
Clinical Judgment (25)	47,436–60,652	**18,125–22,159**	**8,972–11,366**
Clinical Judgment (50)	**11,890–15,621**	**3,175–4,146**	**301–583**
Clinical Judgment (75)	**1,324–2,136**	***Dominant***	***Dominant***
PCR Test (90/95)*	**3,628–4,988**	***Dominant***	***Dominant***
PCR Test (90/100)	**2,789–3,750**	***Dominant***	***Dominant***
PCR Test (95/100)	**2,463–3,142**	***Dominant***	***Dominant***
PCR Test (90/95)+CJ (25)	**23,745–26,472**	**9,311–9,902**	**3,446–4,164**
PCR Test (90/95)+CJ (50)	**20,588–29,728**	**7,094–10,090**	**6,472–9,101**
PCR Test (90/95)+CJ (75)	**20,326–27,346**	**21,115–26,993**	**19,304–25,901**
PCR Test (90/100)+CJ (25)	**19,423–26,903**	**8,901–9,442**	**2,973–4,390**
PCR Test (90/100)+CJ (50)	**20,453–27,160**	**7,303–9,532**	**3,118–4,160**
PCR Test (90/100)+CJ (75)	**21,115–26,993**	**7,242–9,784**	**3,027–4,139**
PCR Test (95/100)+CJ (25)	**18,466–25,825**	**6,491–8,675**	**3,062–3,314**
PCR Test (95/100)+CJ (50)	**18,581–24,773**	**6,472–9,010**	**2,775–3,285**
PCR Test (95/100)+CJ (75)	**19,304–25,901**	**6,741–8,780**	**2,629–3,699**
Point-of-Care Test (25/95)	**16,469–27,039**	**6,874–8,395**	**3,155–4,230**
Point-of-Care Test (50/95)	**6,623–8,804**	**1,491–1,888**	***Dominant***
Point-of-Care Test (75/95)	**3,219–4,145**	***Dominant***	***Dominant***
Point-of-Care Test (25/95)+CJ (25)	**35,678–44,618**	**13,479–16,596**	**6,790–8,967**
Point-of-Care Test (25/95)+CJ (50)	**15,794–21,438**	**5,723–7,582**	**1,912–2,597**
Point-of-Care Test (25/95)+CJ (75)	**7,103–9,563**	**1,506–1,999**	***Dominant***
Point-of-Care Test (50/95)+CJ (25)	**22,777–31,938**	**8,294–11,270**	**3,428–4,805**
Point-of-Care Test (50/95)+CJ (50)	**15,794–21,438**	**5,723–7,582**	**1,912–2,597**
Point-of-Care Test (50/95)+CJ (75)	**7,103–9,563**	**1,506–1,999**	***Dominant***
Point-of-Care Test (75/95)+CJ (25)	**16,316–21,394**	**5,492–7,410**	**1,871–2,511**
Point-of-Care Test (75/95)+CJ (50)	**10,183–14,170**	**2,899–4,208**	**478–555**
Point-of-Care Test (75/95)+CJ (75)	**5,741–7,635**	**826–1,079**	***Dominant***

Comparator: Do nothing.

† (Sensitivity).

*(Sensitivity/Specificity).

Bold Text: Strategy is cost effective (ICER versus Do Nothing is <$50,000 per QALY).

Bold and Italic Text: Strategy is economically dominant (costs less and is more effective than Do Nothing).

### Comparison of All Strategies

For adults, clinical judgment emerged as the most cost-effective strategy when influenza made up 30% of seasonal ILI cases from the societal perspective; this was followed by PCR (ICER: $50,864/QALY) and point-of-care testing (ICER: $342,873/QALY compared to PCR). From the third-party payor perspective and societal perspective at 10% influenza, the do-nothing strategy was the best, followed by clinical judgment (ICER ≤$148,358/QALY), point-of-care (ICER: ≤$202,127/QALY) and PCR testing (ICER: ≤$94,165/QALY compared to point-of-care). For pandemic influenza, clinical judgment (≥20% influenza) dominated from the societal perspective, followed by doing nothing, PCR (ICER: $37,286/QALY), then point-of-care testing (dominated by PCR). From the third-party payor perspective, the do nothing strategy emerged as the most cost-effective, then clinical judgment ($47,841/QALY), and point-of-care testing ($202,124/QALY compared to clinical judgment).

Among older adults (65+ years old), PCR testing emerged as the most cost-effective strategy from both perspectives, dominating all others in both seasonal and pandemic scenarios. From the societal perspective, when ≥20% of cases were influenza, clinical judgment followed PCR as the next most cost-effective, then by point-of-care (≤$215,650/QALY compared to clinical judgment) and point-of-care plus clinical judgment (≤$14,998/QALY compared to point-of-care alone). From the third-party payor perspective, PCR testing was followed by the do nothing strategy, clinical judgment ($16,545/QALY compared to doing nothing), then point-of-care testing ($173,895/QALY compared to clinical judgment) for seasonal influenza. In a pandemic influenza scenario, PCR testing dominated, followed by clinical judgment, and point-of-care testing (≤$287,530/QALY compared to clinical judgment).

## Discussion

Our study results suggest that for healthy younger adults (ages 20 to 64) from the third-party payor perspective, antiviral costs outweigh the potential benefits of testing or antiviral use as long as the virus has the same virulence as seasonal influenza. From the societal perspective, PCR testing and highly sensitive clinical judgment are cost-effective when influenza constitutes ≥20% of ILI cases. For more virulent circulating virus strains or for higher-risk patients, clinical judgment ≥50% sensitive, PCR, point-of-care, and point-of-care in combination with highly sensitive clinical judgment were cost-effective (societal perspective) but only when influenza constitutes at least 20% of all ILI cases. While clinicians may be tempted to do so, treating all younger adult ILI patients with antivirals is unlikely to be a cost-effective approach.

Findings were quite different for older adults (65+ years old). Routine PCR testing of ILI cases seems cost-effective when the probability of ILI being influenza is at least 10%. This presumes that PCR is available at the time of the clinic visit, results are rapidly available, and, if the test is positive, antiviral medications are initiated within 48 hours, which may not be feasible in many settings. Moreover, this assumed that testing every infected person would not overwhelm laboratory facilities. Clinical judgment ≥50% sensitive also appears to be cost-effective in both seasonal and pandemic scenarios. Point-of-care testing in combination with clinical judgment and using PCR to confirm clinical judgment were cost-effective when ≥20% of ILI was influenza. All testing strategies were cost-effective from the societal perspective. Treating all older adults with antivirals may be a cost-effective option as well.

For patients at much higher risk for complications, employing PCR emerged as the most cost-effective option with clinical judgment being the closest competitor but only when judgment sensitivity reached or exceeded 50%. Complication risk may also be elevated in pandemic scenarios with a more virulent circulating strain. In a pandemic scenario, prescribing antivirals to all symptomatic patients may be warranted for older adults but not younger adults.

The performance of clinical judgment (as well as that of other testing strategies) depends on the definition of ILI. The more lenient the definition of ILI, the lower the probability of ILI being influenza will be. Our study assumed the current Centers for Disease Control and Prevention (CDC) definition of ILI: fever ≥100°F and cough and/or sore throat, in the absence of a known cause other than influenza [15], [16]. The optimal influenza testing strategy may be different depending on when during an epidemic a patient presents with ILI. As our study has shown, the economic value of each strategy is sensitive to the proportion of ILI that is influenza. Early in an epidemic, this proportion may be rather low. However, this proportion increases as the epidemic reaches its peak and then starts to decrease. Therefore, real-time awareness of local epidemiologic data (e.g., percent ILI that is influenza), may help decision making [10], [17].

Our results are consistent with studies suggesting that neuraminidase inhibitors have modest efficacy and should be optional for healthy adults during typical influenza seasons yet recommended for high risk adults and epidemic situations with more virulent strains [17], [18], [19]. However, not all studies are in agreement, with some showing oseltamivir use to be cost-effective for healthy adults, children, elderly, and individuals at increased risk for complications [20]. Sintchenko et al suggested that low-risk patients with ILI should be tested before treated with antivirals and that high-risk patients would benefit from prompt treatment [21]. Our study suggests that for healthy younger adults doing nothing is favorable until influenza constitutes 20% or more of ILI cases, when testing becomes favorable. By contrast, testing is consistently more cost-effective than doing nothing for older adults.

The CDC state that most persons with uncomplicated H1N1 influenza do not need testing and notes that when a decision is made to use antiviral treatment for influenza, treatment should be initiated as soon as possible without waiting for influenza test results [22]. Indeed, antiviral treatment is more effective when administered as early as possible in the course of illness. CDC has created an algorithm for adults with ILI to assist in guidance as to who is at higher risk for influenza and its complications [23]. CDC also has recommendations for antiviral usage [24]. Our analysis adds to the CDC guidelines by showing the importance of either highly sensitive clinical judgment or PCR.

Unfortunately, clinical diagnosis of influenza is problematic. In the Rational Clinical Examination Series, the authors reported that clinical findings identify patients with ILI but are not particularly useful for confirming or excluding the diagnosis of influenza [10]. Factors decreasing the likelihood of influenza included the absence of fever, cough, or nasal congestion, findings with likelihood ratios (LR) <0.5. In studies limited to patients aged 60 years or older, the combination of fever, cough, and acute onset had the highest LR of 5.4.

The Infectious Disease Society of America (IDSA) released guidelines in 2009 for seasonal influenza that indicate which persons should be tested for influenza if the result will influence clinical management, including initiation of antiviral medications [25]. IDSA recommends treatment for seasonal influenza for persons who meet the specified criteria, including those with laboratory-confirmed or highly suspected influenza virus infection at high risk of developing complications and are within 48 hours after symptom onset. According to IDSA, treatment should be considered for outpatients with laboratory-confirmed or highly suspected influenza virus infection who are not at increased risk of complications, whose onset of symptoms is less than 48 hours before presentation, and who wish to shorten the duration of illness and further reduce their relatively low risk of complications. IDSA revisited these guidelines in light of the pandemic.

### Limitations

No computer model can fully represent every single possible influenza event and outcome. Models, by definition, are simplifications of real life. While in our study, we explored some possible higher-risk patient scenarios, fully representing the wide range of possible increases in hospitalization risk and mortality is difficult. The impact of co-morbidities can be variable and unexpected, which may increase their corresponding resource use (e.g., mechanical ventilation). This risk varies depending on the underlying condition (asthma vs. pregnancy vs. cardiovascular disease), the number of comorbidities, and the timing of antiviral initiation. Clear definitions of high risk groups are evolving as pandemics progress; for example, obesity has been considered in some studies to confer increased risk while HIV infection has not conferred as much increased risk as initially thought. There is a dearth of data on how delaying administration of antivirals will reduce antiviral efficacy, especially when patients present to the clinic or emergency room at different stages of infection. To remain conservative about the benefits of antivirals, our model did not include the potential ability of antivirals to reduce transmission. It can be challenging to model transmission effects on a patient presenting to a clinic or emergency room, who may have any number of contact rates and patterns before and after the visit. Moreover, there remains debate over the efficacy of antivirals in preventing transmission.

### Conclusions

Our study delineated the conditions under which different testing and antiviral strategies may be cost-effective. For healthy adults aged 20 to 64 years with seasonal influenza, none of the tested strategies were found to be cost-effective from the third-party payor perspective. When hospitalization risk and mortality were doubled, using clinical judgment (≥50% sensitive) to guide antiviral initiation emerged as the most cost-effective option with PCR testing being the closest competitor but only when at least 20% of ILI cases were influenza. Among older adults (65+ years old), employing PCR to guide antiviral initiation emerged as the most cost-effective option with the closest competitor being clinical judgment when judgment sensitivity was at least 50%. Treating all ILI patients with antivirals appeared to be cost-effective only in older adults.

## Supporting Information

Table S1 Incremental cost-effectiveness ratios (in $US per quality-adjusted life-years) of different approaches to patients aged 20 to 64 years with influenza-like illness (ILI) from the third-party payor perspective.
(0.10 MB DOC)

Table S2 Incremental cost-effectiveness ratios (in $US per quality-adjusted life-years) of different approaches to patients aged 65 to 85 years with influenza-like illness (ILI) from the third-party payor perspective.
(0.21 MB DOC)
