# Supplementary material for: To Test or to Treat? An Analysis of Influenza Testing and Antiviral Treatment Strategies Using Economic Computer Modeling
Source: PLoS One. 2010 Jun 23;5(6):e11284. doi: 10.1371/journal.pone.0011284 (PMC2890406; doi:10.1371/journal.pone.0011284)
Supplement: Table S1 — Incremental cost-effectiveness ratios (in $US per quality-adjusted life-years) of different approaches to patients aged 20 to 64 years with influenza-like illness (ILI) from the third-party payor perspective. (0.10 MB DOC) [file pone.0011284.s001.doc]

TABLE S1

|  | **Probability of ILI being Influenza** | | |
| --- | --- | --- | --- |
| **Strategy** | **10%** | **20%** | **30%** |
| *Baseline Seasonal Influenza Hospitalization Risk and Mortality* | | | |
| Treat all with Antivirals | Do Nothing | 485,657 – 586,314 | 186,881 – 217,551 |
| Clinical Judgment (25)† | Do Nothing | Do Nothing | Do Nothing |
| Clinical Judgment (50) | Do Nothing | 494,416 – 548,374 | 189,015 – 209,843 |
| Clinical Judgment (75) | 304,661 – 375,012 | 112,677 – 138,610 | 78,225 – 84,236 |
| PCR Test (90/95)* | 210,735 – 283,306 | 133,747 – 147,084 | 99,757 – 120,599 |
| PCR Test (90/100) | 202,555 – 207,361 | 112,122 – 130,354 | 90,770 – 100,013 |
| PCR Test (95/100) | 185,110 – 208,647 | 116,522 – 122,312 | 88,137 – 90,757 |
| PCR Test (90/95) + CJ (25) | Do Nothing | 471,123 – 857,015 | 309,579 – 401,633 |
| PCR Test (90/95) + CJ (50) | Do Nothing | 969,670 – 2,697,988 | 283,215 – 341,330 |
| PCR Test (90/95) + CJ (75) | Do Nothing | 826,131 – 910,265 | 282,471 – 372,160 |
| PCR Test (90/100) + CJ (25) | Do Nothing | 673,177 – 858,328 | 277,799 – 277,579 |
| PCR Test (90/100) + CJ (50) | Do Nothing | 719,767 – 1,217,744 | 296,911 – 335,417 |
| PCR Test (90/100) + CJ (75) | Do Nothing | 784,861 – 994,763 | 337,825 – 392,7800 |
| PCR Test (95/100) + CJ (25) | Do Nothing | 435,917 – 525,626 | 310,571 – 435,358 |
| PCR Test (95/100) + CJ (50) | Do Nothing | 567,996 – 619,082 | 244,285 – 323,209 |
| PCR Test (95/100) + CJ (75) | Do Nothing | 581,755 – 828,280 | 298,502 – 304,800 |
| Point-of-Care Test (25/95) | 849,556 – 997,358 | 266,731 – 358,977 | 272,831 – 322,543 |
| Point-of-Care Test (50/95) | 313,600 – 417,942 | 183,375 – 226,543 | 139,994 – 151,646 |
| Point-of-Care Test (75/95) | 262,842 – 266,510 | 123,663 – 141,856 | 91,446 – 111,997 |
| Point-of-Care Test (25/95) + CJ (25) | Do Nothing | Do Nothing | 756,667 – 1,144,269 |
| Point-of-Care Test (25/95) + CJ (50) | Do Nothing | 340,489 – 504,602 | 244,626 – 262,133 |
| Point-of-Care Test (25/95) + CJ (75) | 515,324 – 667,671 | 202,184 – 253,089 | 137,171 – 137,499 |
| Point-of-Care Test (50/95) + CJ (25) | Do Nothing | 876,156 – 1,345,743 | 328,747 – 394,001 |
| Point-of-Care Test (50/95) + CJ (50) | 1,944,613 – 2,209,253 | 340,489 – 504,602 | 203,088 – 226,217 |
| Point-of-Care Test (50/95) + CJ (75) | 509,769 – 535,382 | 177,843 – 211,243 | 122,116 – 135,559 |
| Point-of-Care Test (75/95) + CJ (25) | Do Nothing | 533,989 – 629,120 | 328,748 – 394,001 |
| Point-of-Care Test (75/95) + CJ (50) | 2,075,665 – 2,947,007 | 285,441 – 330,652 | 164,651 – 192,729 |
| Point-of-Care Test (75/95) + CJ (75) | 420,377 – 665,562 | 173,114 – 200,116 | 117,221 – 119,575 |
| *Pandemic or High Risk Patients (2x Seasonal Influenza Hospitalization Risk and Mortality)* | | | |
| Treat all with Antivirals | 181,431 – 225,462 | 87,258 – 101,010 | 69,894 – 69,900 |
| Clinical Judgment (25)† | Do Nothing | 905,622 – 2,572,853 | 245,830 – 441,958 |
| Clinical Judgment (50) | 496,681 – 1,353,821 | 132,897 – 134,129 | 74,562 – 88,892 |
| Clinical Judgment (75) | 76,115 – 122,336 | 42,923 – 59,158 | **30,098 – 35,000** |
| PCR Test (90/95)* | 110,521 – 125,601 | 55,543 – 69,150 | 43,181 – 52,427 |
| PCR Test (90/100) | 92,657 – 120,295 | 52,387 – 58,763 | **39,210 – 50,035** |
| PCR Test (95/100) | 85,796 – 106,707 | 50,246 – 58,289 | **38,109 – 46,432** |
| PCR Test (90/95) + CJ (25) | Do Nothing | 161,630 – 262,156 | 143,503 – 150,055 |
| PCR Test (90/95) + CJ (50) | 1,104,201 – 1,856,861 | 186,228 – 240,229 | 121,223 – 125,056 |
| PCR Test (90/95) + CJ (75) | 626,296 – 2,653,755 | 190,215 – 234,940 | 114,136 – 126,702 |
| PCR Test (90/100) + CJ (25) | 1,499,057 – 1,661,532 | 184,526 – 226,061 | 136,006 – 138,541 |
| PCR Test (90/100) + CJ (50) | 854,704 – 1,214,574 | 159,929 – 261,287 | 112,321 – 155,408 |
| PCR Test (90/100) + CJ (75) | 547,383 – 1,844,658 | 207,748 – 298,600 | 101,444 – 139,208 |
| PCR Test (95/100) + CJ (25) | 470,524 – 135,576 | 141,757 – 183,626 | 97,839 – 113,001 |
| PCR Test (95/100) + CJ (50) | 506,525 – 851,104 | 188,655 – 232,268 | 97,416 – 103,640 |
| PCR Test (95/100) + CJ (75) | 614,000 – 1,167,947 | 155,989 – 210,237 | 114,209 – 117,332 |
| Point-of-Care Test (25/95) | 221,218 – 346,605 | 132,345 – 183,032 | 96,979 – 105,438 |
| Point-of-Care Test (50/95) | 161,055 – 190,928 | 75,695 – 91,296 | 56,712 – 70,223 |
| Point-of-Care Test (75/95) | 106,938 – 142,140 | 55,510 – 70,781 | **39,246 – 51,189** |
| Point-of-Care Test (25/95) + CJ (25) | Do Nothing | 291,052 – 598,607 | 168,489 – 195,005 |
| Point-of-Care Test (25/95) + CJ (50) | 435,252 – 745,254 | 142,732 – 145,656 | 86,489 – 112,413 |
| Point-of-Care Test (25/95) + CJ (75) | 158,366 – 198,511 | 83,110 – 99,804 | 52,983 – 62,141 |
| Point-of-Care Test (50/95) + CJ (25) | 1,037,606 – 1,957,182 | 220,655 – 316,968 | 115,039 – 158,722 |
| Point-of-Care Test (50/95) + CJ (50) | 363,789 – 384,328 | 125,958 – 143,661 | 80,256 – 90,683 |
| Point-of-Care Test (50/95) + CJ (75) | 156,105 – 203,779 | 76,426 – 83,153 | 51,478 – 67,873 |
| Point-of-Care Test (75/95) + CJ (25) | 501,339 – 715,201 | 161,953 – 172,963 | 90,891 – 98,779 |
| Point-of-Care Test (75/95) + CJ (50) | 243,241 – 329,603 | 113,757 – 138,750 | 70,122 – 85,622 |
| Point-of-Care Test (75/95) + CJ (75) | 150,704 – 166,733 | 69,097 – 86,970 | 48,014 – 60,215 |

Comparator: Do nothing.

† (Sensitivity)

* (Sensitivity/Specificity)

Bold Text: Strategy is cost effective (ICER versus Do Nothing is <$50,000 per QALY)

Bold and Italic Text: Strategy is economically dominant (costs less and is more effective than Do Nothing)
