# Supplementary material for: To Test or to Treat? An Analysis of Influenza Testing and Antiviral Treatment Strategies Using Economic Computer Modeling
Source: PLoS One. 2010 Jun 23;5(6):e11284. doi: 10.1371/journal.pone.0011284 (PMC2890406; doi:10.1371/journal.pone.0011284)
Supplement: Table S2 — Incremental cost-effectiveness ratios (in $US per quality-adjusted life-years) of different approaches to patients aged 65 to 85 years with influenza-like illness (ILI) from the third-party payor perspective. (0.21 MB DOC) [file pone.0011284.s002.doc]

TABLE S2

|  | **Probability of ILI being Influenza** | | |
| --- | --- | --- | --- |
| **Strategy** | **10%** | **20%** | **30%** |
| *Baseline Seasonal Influenza Hospitalization Risk and Mortality* | | | |
| Treat all with Antivirals | 43,346 – 66,474 | **8,434 – 11,466** | ***Dominant*** |
| Clinical Judgment (25)† | 239,721 – 884,952 | 77,564 – 91,844 | **27,584 – 42,138** |
| Clinical Judgment (50) | 38,889 – 72,626 | **9,133 – 10,044** | ***Dominant*** |
| Clinical Judgment (75) | **1,466 – 1,825** | ***Dominant*** | ***Dominant*** |
| PCR Test (90/95)* | **9,604 – 14,173** | ***Dominant*** | ***Dominant*** |
| PCR Test (90/100) | **6,703 -8,867** | ***Dominant*** | ***Dominant*** |
| PCR Test (95/100) | **5,349 – 7,176** | ***Dominant*** | ***Dominant*** |
| PCR Test (90/95) + CJ (25) | 81,265 – 120,399 | **23,606 – 32,606** | **7,031 – 8,264** |
| PCR Test (90/95) + CJ (50) | 84,830 – 135,860 | **24,675 – 35,616** | **8,211 – 10,938** |
| PCR Test (90/95) + CJ (75) | 83,205 – 112,208 | **25,863 – 34,030** | **7,594 – 11,299** |
| PCR Test (90/100) + CJ (25) | 70,393 – 127,511 | **21,009 – 34,788** | **7,524 – 10,482** |
| PCR Test (90/100) + CJ (50) | 86,295 – 126,626 | **23,750 – 35,750** | **8,337 – 10,890** |
| PCR Test (90/100) + CJ (75) | 92,622 – 108,647 | **24,550 – 33,926** | **7,848 – 10,546** |
| PCR Test (95/100) + CJ (25) | 69,761 – 125,674 | **20,463 – 27,803** | **6,842 – 8,271** |
| PCR Test (95/100) + CJ (50) | 72,522 – 106,080 | **24,102 – 30,175** | **6,034 – 8,179** |
| PCR Test (95/100) + CJ (75) | 74,763 – 108,756 | **21,330 – 28,843** | **6,072 – 8,442** |
| Point-of-Care Test (25/95) | 57,261 – 90,951 | **18,596 – 24,750** | **7,487 – 10,213** |
| Point-of-Care Test (50/95) | **19,945 – 33,595** | **1,613 - 1,831** | ***Dominant*** |
| Point-of-Care Test (75/95) | **7,926 – 10,380** | ***Dominant*** | ***Dominant*** |
| Point-of-Care Test (25/95) + CJ (25) | 137,022 – 235,145 | 50,525 – 71,859 | **22,513 – 29,453** |
| Point-of-Care Test (25/95) + CJ (50) | 63,714 – 85,454 | **19,375 – 26,267** | **3,712 – 4,908** |
| Point-of-Care Test (25/95) + CJ (75) | **23,968 – 33,268** | **1,637 – 2,314** | ***Dominant*** |
| Point-of-Care Test (50/95) + CJ (25) | 94,695 – 130,535 | **28,350 – 39,945** | **9,848 – 12,881** |
| Point-of-Care Test (50/95) + CJ (50) | 49,215 – 71,166 | **11,505 – 16,674** | **325 - 432** |
| Point-of-Care Test (50/95) + CJ (75) | **21,040 – 27,494** | **282 - 349** | ***Dominant*** |
| Point-of-Care Test (75/95) + CJ (25) | 61,176 – 86,00 | **16,489 – 23,117** | **3,345 – 4,151** |
| Point-of-Care Test (75/95) + CJ (50) | **35,943 – 50,951** | **7,353 – 9,312** | ***Dominant*** |
| Point-of-Care Test (75/95) + CJ (75) | **18,219 -23,300** | ***Dominant*** | ***Dominant*** |
| *Pandemic or High Risk Patients (2x Seasonal Influenza Hospitalization Risk and Mortality)* | | | |
| Treat all with Antivirals | **8,205 – 10,600** | ***Dominant*** | ***Dominant*** |
| Clinical Judgment (25) | 39,716 – 51,709 | **13,888 – 17,987** | **4,223 – 5,271** |
| Clinical Judgment (50) | **8,326 – 10,392** | ***Dominant*** | ***Dominant*** |
| Clinical Judgment (75) | ***Dominant*** | ***Dominant*** | ***Dominant*** |
| PCR Test (90/95)* | ***Dominant*** | ***Dominant*** | ***Dominant*** |
| PCR Test (90/100) | ***Dominant*** | ***Dominant*** | ***Dominant*** |
| PCR Test (95/100) | ***Dominant*** | ***Dominant*** | ***Dominant*** |
| PCR Test (90/95) + CJ (25) | **16,173 – 22,398** | **3,642 – 4,186** | ***Dominant*** |
| PCR Test (90/95) + CJ (50) | **16,622 – 20,818** | **3,628 – 4,848** | ***Dominant*** |
| PCR Test (90/95) + CJ (75) | **15,552 – 21,557** | **3,656 – 4,761** | ***Dominant*** |
| PCR Test (90/100) + CJ (25) | **14,691 – 23,595** | **3,492 – 4,707** | ***Dominant*** |
| PCR Test (90/100) + CJ (50) | **15,778 – 21,636** | **3,400 – 4,511** | ***Dominant*** |
| PCR Test (90/100) + CJ (75) | **16,411 – 21,998** | **3,306 – 4,807** | ***Dominant*** |
| PCR Test (95/100) + CJ (25) | **14,790 – 21,261** | **2,579 – 3,835** | ***Dominant*** |
| PCR Test (95/100) + CJ (50) | **15,359 – 19,250** | **2,964 – 3,725** | ***Dominant*** |
| PCR Test (95/100) + CJ (75) | **15,223 – 21,232** | **2,977 – 3,971** | ***Dominant*** |
| Point-of-Care Test (25/95) | **13,728 – 19,122** | **2,592 – 3,715** | ***Dominant*** |
| Point-of-Care Test (50/95) | **2,773 – 3,695** | ***Dominant*** | ***Dominant*** |
| Point-of-Care Test (75/95) | ***Dominant*** | ***Dominant*** | ***Dominant*** |
| Point-of-Care Test (25/95) + CJ (25) | **30,313 – 42,710** | **9,437 – 12,937** | **2,800 – 3,872** |
| Point-of-Care Test (25/95) + CJ (50) | **12,690 – 16,864** | **1,709 – 2,292** | ***Dominant*** |
| Point-of-Care Test (25/95) + CJ (75) | **3,227 – 4,586** | ***Dominant*** | ***Dominant*** |
| Point-of-Care Test (50/95) + CJ (25) | **17,834 – 26,327** | **4,338 – 5,803** | ***Dominant*** |
| Point-of-Care Test (50/95) + CJ (50) | **8,755 – 12,974** | **207 - 288** | ***Dominant*** |
| Point-of-Care Test (50/95) + CJ (75) | **2,663 – 3,556** | ***Dominant*** | ***Dominant*** |
| Point-of-Care Test (75/95) + CJ (25) | **12,289 – 16,745** | **1,688 – 2,270** | ***Dominant*** |
| Point-of-Care Test (75/95) + CJ (50) | **6,615 – 9,620** | ***Dominant*** | ***Dominant*** |
| Point-of-Care Test (75/95) + CJ (75) | **1,931 – 2,644** | ***Dominant*** | ***Dominant*** |

Comparator: Do nothing.

† (Sensitivity)

* (Sensitivity/Specificity)

Bold Text: Strategy is cost effective (ICER versus Do Nothing is <$50,000 per QALY)

Bold and Italic Text: Strategy is economically dominant (costs less and is more effective than Do Nothing)
